# Supplementary figures and images for: Mid-Devensian climate and landscape in England: new data from Finningley, South Yorkshire
Source: R Soc Open Sci. 2019 Jul 10;6(7):190577. doi: 10.1098/rsos.190577 (PMC6689596; doi:10.1098/rsos.190577)

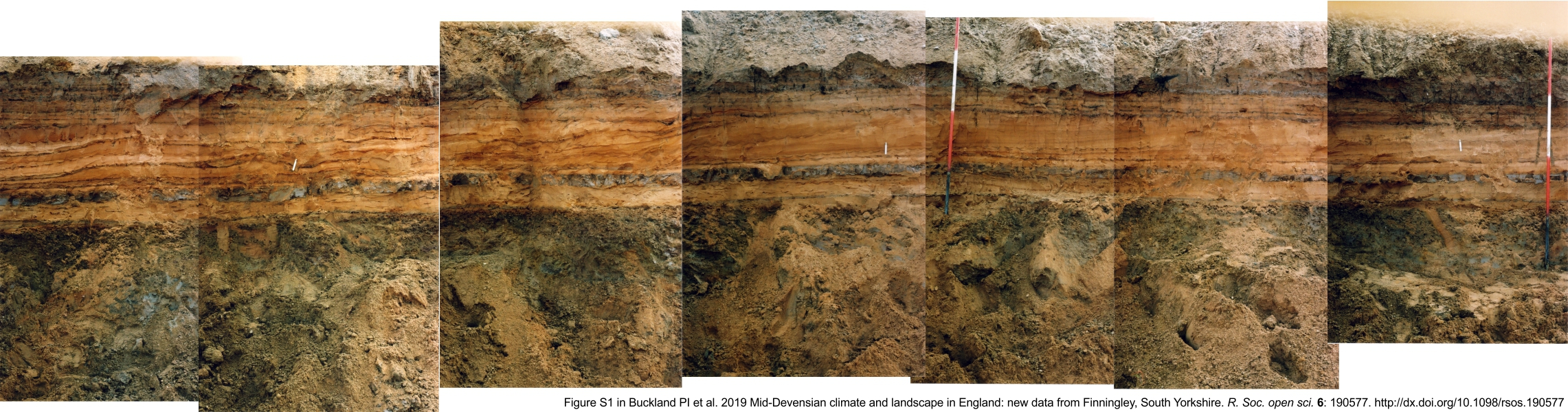

Supplement: Figure S1 [file rsos190577supp1.jpg]

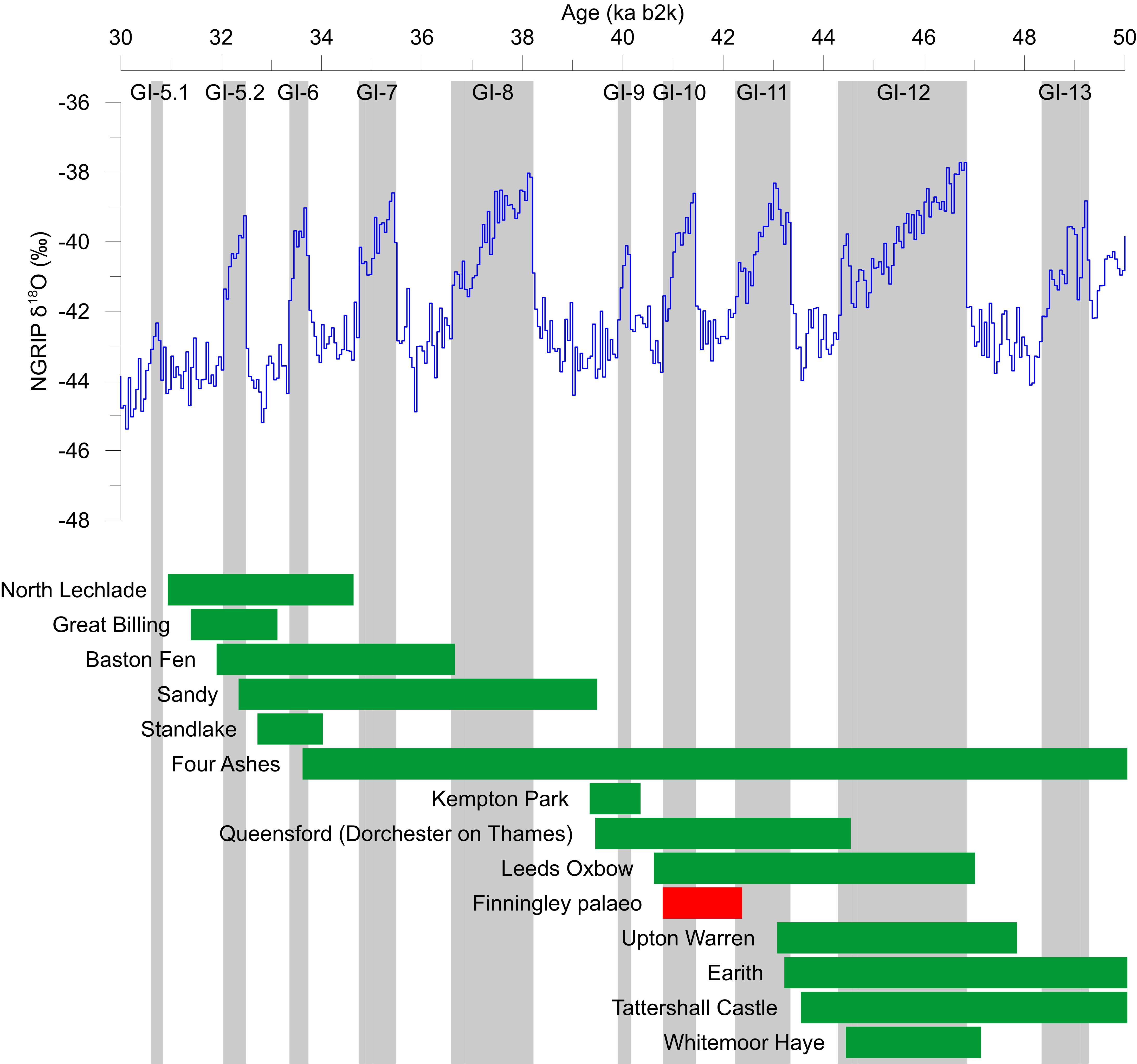

Supplement: Figure S3. Site radiocarbon dates compared with Greenland Ice Core data [file rsos190577supp3.jpg]
